# How coordinated link sharing behavior and partisans’ narrative framing fan the spread of COVID-19 misinformation and conspiracy theories

**DOI:** 10.1007/s13278-022-00948-y

**Published:** 2022-08-20

**Authors:** Anatoliy Gruzd, Philip Mai, Felipe Bonow Soares

**Affiliations:** Ted Rogers School of Management, Social Media Lab, Toronto Metropolitan University, Toronto, Canada

**Keywords:** Coordinated link sharing behavior, Covid-19, Misinformation, Conspiracy theories, Social media, Facebook

## Abstract

This study examines the presence and role of Coordinated Link Sharing Behavior (CLSB) on Facebook around the “America’s Frontline Doctors” press conference, and the promotion of several unproven conspiracy theories including the false assertion that hydroxychloroquine is a “cure” for COVID-19 by Dr. Stella Immanuel, one of the doctors who took part in the press conference. We collected 7,737 public Facebook posts mentioning Stella Immanuel using CrowdTangle and then applied the specialized program CooRnet to detect CLSB among Facebook public pages, groups and verified profiles. Finally, we used a mixed-method approach consisting of both network and content analysis to examine the nature and scope of the detected CLSB. Our analysis shows how Facebook accounts engaged in CLSB to fuel the spread of misinformation. We identified a coalition of Facebook accounts that engaged in CLSB to promote COVID-19 related misinformation. This coalition included US-based pro-Trump, QAnon, and anti-vaccination accounts. In addition, we identified Facebook accounts that engaged in CLSB in other countries, such as Brazil and France, that primarily promoted hydroxychloroquine, and some accounts in African countries that criticized the government's pandemic response in their countries.

## Introduction

The COVID-19 pandemic is proving to be a powerful rallying cry for conspiracy theorists and fringe groups from around the world (Allington et al. 2021; Bertin et al. 2020; Bruns et al. 2020). It has brought together everyone from wellness influencers, to anti-vaxxers, to anti-immigrant groups to white supremacists. This paper will examine one example of this trend. In July of 2020, a group of doctors who called themselves “America’s Frontline Doctors,” convened a press conference in Washington DC to tout several unproven conspiracy theories about COVID-19, including the false assertion that hydroxychloroquine is a “cure” for COVID-19. Dr. Stella Immanuel, one of the doctors on the panel and is the key figure of our case study, made a number of statements in favor of using hydroxychloroquine to treat COVID-19, citing her own experience of using the drug to treat patients. She also called in question several trials showing the drug’s ineffectiveness against COVID-19 because, in her words, they were done by “fake science.” This is the same antimalarial drug promoted by President Trump in early 2020 (Grady et al. 2020).

The live stream and video recordings of the press conference went viral on social media and were watched by millions of people around the world on Facebook, YouTube, Twitter and other social media platforms (Passantino and Darcy 2020). A few hours after the press conference was live streamed, social media platforms began fact-checking and removing videos of the press conference from their platforms for sharing claims about hydroxychloroquine which were against existing medical evidence at that time (Gallagher 2020; Jacqueline 2020). The press conference and the subsequent viral spread of its video recordings across social media is an illustrative example of how COVID-related misinformation can spread and reach millions in the matter of hours and even minutes.

Central to our case study, Dr. Stella Immanuel rose to prominence after her statements in support of hydroxychloroquine received praise from President Trump, and after it was reported that she had a history of attributing medical conditions to non-scientific causes such as witches and demons (Smith 2020). Dr. Immanuel quickly became the focus of media attention and a popular topic of discussion across social media platforms. Considering Dr. Immanuel’s newly discovered notoriety, we decided to examine who on Facebook (the largest social media platform in the world) mentioned the doctor immediately after the press conference and whether these users used this as an opportunity to push and amplify COVID-19 misinformation in a coordinated way.

Specifically, we examined data to look for online actors who were engaged in coordinated links sharing behavior (CLSB), by sharing the same links within a few minutes or even seconds apart. The reason for focusing on CLSB is because CLSB and coordinated sharing behavior (more broadly) have shown to be linked to the propagation of problematic content such as misinformation and conspiracy theories (Graham et al. 2020; Giglietto et al. 2020b, 2020c). Furthermore, Papakyriakopoulos et al. ( 2020) showed that highly active coordinated accounts can manipulate political discussions and create bias in recommender systems to make social media content appear to be more popular than it actually is.

While this type of behavior is often associated with inauthentic accounts such as bots and fake profiles, ‘authentic’ accounts may also engage in coordinated behavior (Nizzoli et al. 2021). For example, harmful authentic accounts such as online trolls may engage in a coordinated action to spread hate speech and attack individuals or groups with whom they disagree (Bradshaw and Howard 2017). In one study, Tandoc et al. (2021) documented how women journalists were systematically harassed on social media by so-called “troll armies.”

Although research on misinformation is a rapidly growing field (Righetti 2021), the detection and analysis of coordinated sharing behavior on social media is an understudied area. This is mostly due to the lack of data and tools to detect coordinated behavior on social media automatically and reliably. In this study, we used a recently developed, specialized R program called CooRnet designed to detect CLSB among Facebook entities (Giglietto et al. 2020a). By using CooRnet to analyze and detect CLSB, this paper offers empirical evidence to further understand the practice of CLSB in the context of COVID-19 discussions and link sharing on Facebook.

The following section will review the previous work in this area as it pertains to our research questions.

## Literature review and research questions

### Coordinated link sharing behavior (CLSB)

Most studies that have explored coordinated sharing behavior have been focused on political events. Giglietto et al. (2020b) analyzed Facebook posts about the 2018 Italian general election and 2019 European election and demonstrated that rapid sharing of URLs by the same group of entities was associated with coordinated and inauthentic behavior on Facebook. The authors identified that the Facebook accounts engaged in CLSB aimed to manipulate the media and public opinion during the elections. Similarly, Nizzoli et al. (2021) analyzed Twitter data from the 2019 UK general election and found that many coordinated networks included a higher degree of automation and several accounts that were later suspended. Yu (2021) analyzed Facebook posts about the 2019 Philippine Midterm Elections and found that most of the media (URLs, photos, and videos) shared in a coordinated manner were no longer available (posts likely removed by Facebook, and links to websites that no longer exist), suggesting that coordinated accounts were engaged in sharing problematic content.

Other investigations of CLSB have focused on COVID-19. Graham et al. (2020) explored coordinated behavior on Twitter in discussions about COVID-19. The authors found clusters of coordinated accounts promoting the conspiracy theory that COVID-19 was engineered as a bioweapon by China. These coordinated accounts were mostly linked to users expressing conservative and far-right views (Pro-Trump, QAnon, and/or Republican). In another study, Ayers et al. (2021) analyzed CLSB in discussions about face masks on Facebook. The authors found a similar trend that coordinated accounts contributed to the spread of COVID-19 related conspiracy theories on Facebook.

Considering the evidence that at least some COVID-19 related conspiracy theories were shared on social media in a coordinated way, we ask the first question:

*RQ1*: Is there evidence of Coordinated Link Sharing Behavior (CLSB) on Facebook related to discussions about Dr. Stella Immanuel after the press conference with “America’s Frontline Doctors”?

### Political polarization and narrative framing

In addition to exploring a potential link between CLSB and sharing of COVID-19 misinformation and conspiracy theories, we wanted to examine whether CLSB might have been used to spread politically motivated narratives, potentially leading to a high level of political polarization. Indeed, the spread of problematic content online is often influenced by political polarization, fueled by partisan media outlets. Partisan outlets are digital media that provide specific political takes to frame narratives and counter-narratives to support their agendas (Recuero et al. 2020; Kalsnes and Larsson 2021). For example, Recuero et al. (2020) analyzed discussions about the 2018 Brazilian elections on Twitter and found that as polarization between users with opposing points of view increased, so did the influence of partisan outlets linked to misinformation spread.

In some cases, the radicalization of partisan groups can create what Benkler et al. (2018) called asymmetric polarization. The authors studied information consumption and news sharing during the 2018 US Presidential Election and found that groups on the right of the political spectrum were more likely to share content from partisan outlets. Therefore, the idea of asymmetric polarization is used to describe a polarized environment in which one side is strongly associated with misinformation spread. Most studies identified that far-right groups are particularly linked to sharing misinformation (Benkler et al. 2018; Recuero et al. 2020; Kalsnes and Larsson 2021). Nevertheless, even in the context of asymmetric polarization driven by far-right groups, some left-leaning groups also rely on partisan outlets and share misinformation to support their political narratives (Recuero et al. 2020).

These asymmetries are also present in the context of the COVID-19 pandemic. In the USA, republicans were more likely to downplay COVID-19 and believe in misinformation about the pandemic (Calvillo et al. 2020). Partisan motivations, in particular support for Donald Trump, and conservative media consumption were also found among the reasons for Americans to more likely believe in COVID-19 conspiracy theories (Uscinski et al. 2020; Stecula and Pickup 2021).

In Brazil, Recuero et al. (2022) identified that links containing pandemic-related misinformation were mostly shared by right-wing Facebook pages and groups, while fact-checking links were mostly shared by left-wing pages and groups. Brazilians with a right-wing ideology were also found to be more likely to believe in COVID-19 related misinformation (Rossini and Kalogeropoulos 2021). In France, Ward et al. (2020) found that vaccine hesitancy was associated with political radicalization, as both far-left and far-right individuals were more likely to reject COVID-19 vaccines.

Considering, the impact of political polarization on how online actors frame discussions around the pandemic, we ask:

*RQ2*: What is the role of political polarization and partisan outlets in CLSB? Is CLSB a tactic used by either or both right- and left-wing political actors on Facebook to spread narrative and counter-narrative in support of their political views?

### International reach

The rapid and massive spread of COVID-19 related misinformation on social media is a worldwide problem (Tangcharoensathien et al. 2020; Zarocostas 2020). This is because misinformation can easily travel across physical borders (Zarocostas 2020; Bridgman et al. 2021). For example, Nsoesie et al. (2020) found evidence that some misinformation topics spread similarly across several countries, such as the conspiracy theory around 5G and the promotion of natural treatments and unproven drugs for COVID-19. In the analysis of a large dataset of tweets about popular pandemic-related misinformation and conspiracy theories, Bridgman et al. (2021) demonstrated how COVID-19 related misinformation posted by US-based accounts on Twitter spread to Canada, where Canadian users were more likely to encounter misinformation originated in the USA than shared by Canadian sources.

COVID-19 related misinformation originating in the USA also easily crossed in Brazil. After Donald Trump supported the use of hydroxychloroquine, Brazilian President Jair Bolsonaro started promoting the unproven drug for COVID-19 (Casarões and Magalhães 2021). Similarly, a conspiracy theory that encouraged people to take photos and videos of empty hospitals to prove that the pandemic was a hoax started in the USA, but later spread to other countries, including Brazil (Gruzd and Mai 2020).

In yet another example of cross-border misinformation propagation, The Epoch Times, a US-based far-right media outlet, partnered with Tierra Pura, an Argentinian partisan outlet, to translate false stories about COVID-19 to Spanish and Portuguese from English amplifying this content to Latin America and part of Europe (Miguel 2021).

Although the USA is one of the main sources of COVID-19 related misinformation globally, actors from other countries have also shown to be effective in exporting misinformation and conspiracy theories internationally. Dotto and Cubbon (2021) found that foreign anti-vaccine narratives and conspiracy theories reached online social networks in West African countries. While a large share of this social media content was in English and popularized in the USA, the authors also identified misinformation in other languages, such as French and Russian, and originated in other countries. There are also cases of operations controlled by foreign actors, such as the evidence of Russian, Chinese, Turkish and Iranian outlets sharing conspiracy theories and promoting their political agendas in Germany, France, and Spain (Rebello et al. 2020).

To examine the potential presence and impact of cross-border sharing of COVID-19 misinformation and conspiracy theories as related to our case study, we ask:

*RQ3*: Was the CLSB in the studied case constrained to the US-based accounts only? Or, was there a spillover effect, where COVID-19 misinformation originating in the USA was later picked up and shared by Facebook accounts in other countries?

## Method

The data was collected using CrowdTangle, a Meta-owned public content discovery and analysis tool. CrowdTangle tracks public posts published by influential Pages (with more than 25 K likes or followers), Groups (with 95 K members for non-US-based groups or 2 K members for US-based groups), and all verified profiles on Facebook (Fraser 2022). Since Dr. Immanuel was at the core of this case study, we used her full name “Stella Immanuel” as a search query. In total, we retrieved 7,737 public posts shared between July 27 (the date of the press conference) and July 29 (2 days after the press conference) in 2020. Only public posts not yet deleted by users or removed by the platforms at the time of data collection were included in our analysis.

After the data was collected, we used a specialized R program called CooRnet (Giglietto et al. 2020a) to discover what URLs in our dataset were shared most often on Facebook, who shared them and how fast. CooRnet is one of few available tools to detect signs of possible coordinated behavior on Facebook. Most approaches to detect coordinated behavior focus on the publication of the same content from different accounts in a very short time (Nizzoli et al. 2021; Yu 2021). For example, Graham et al. (2020) looked at accounts that retweeted the same messages within one second of each other to detect bot-nets and then 1 min of each other to detect coordinated accounts in general. Similarly, the approach proposed by Weber and Neumann (2021) relies on a temporal analysis combined with other online behaviors, such as retweeting the same message, using the same hashtag, mentioning the same user, and/or sharing the same URL.

We chose to use CooRnet because it has been applied and validated by academic studies and is a suitable tool to work with CrowdTangle data (Giglietto et al. 2020b, 2020c; Ayers et al. 2021). One of the unique features of the program is that it detects when the same URL was shared across multiple Facebook entities (pages, groups and verified profiles) just seconds apart, a sign of potential coordination. The main idea behind this approach is that while it is not uncommon for a group of accounts to share the same URL(s), it is unlikely for them to do so within seconds. The key to this approach is to determine a threshold of what is “unusually” rapid sharing of the same URL(s). To determine this threshold, CooRnet extracted all unique URLs shared in our dataset (899) and separated out 10% of “fastest” shared URLs (i.e., URLs with the shortest time between the first and second time they were shared). The median time it took for these 10% of the fastest shared URLs to reach 50% of the total number of shares is then used to set the threshold for “unusually” fast sharing. Based on our dataset, the threshold was determined to be 71 s.

Next, we used CooRnet to build a “coordinated” link sharing network among Facebook entities from our dataset that met the threshold for engaging in “unusually” rapid sharing (< = 71 s) of the same URL(s). The resulting network consisted of 64,252 ties connecting 1,390 Facebook entities (either a page or group) that shared the same link within a very short period of time, within 71 s as automatically estimated by CooRnet.

We used Gephi (Bastian et al. 2009), a popular program for social network analysis, to identify densely connected clusters of Facebook entities engaged in CLSB using a community detection algorithm.

Lastly, to understand the nature of the discovered clusters, we manually examined the groups, pages and verified profiles within each cluster. As part of this step, we manually verified if the Facebook entities in our dataset were still available on the platform (18 months after the initial data collection) and if they had changed their name or privacy setting. We also manually reviewed the posts and content of the links shared by the entities in the dataset, with a particular focus on the most shared URLs within each major cluster of the resulting CLSB network.

## Results

### Is there evidence of Coordinated Link Sharing Behavior (CLSB) on Facebook related to discussions about Dr. Stella Immanuel after the press conference with “America’s Frontline Doctors”?

**Fig. 1:**
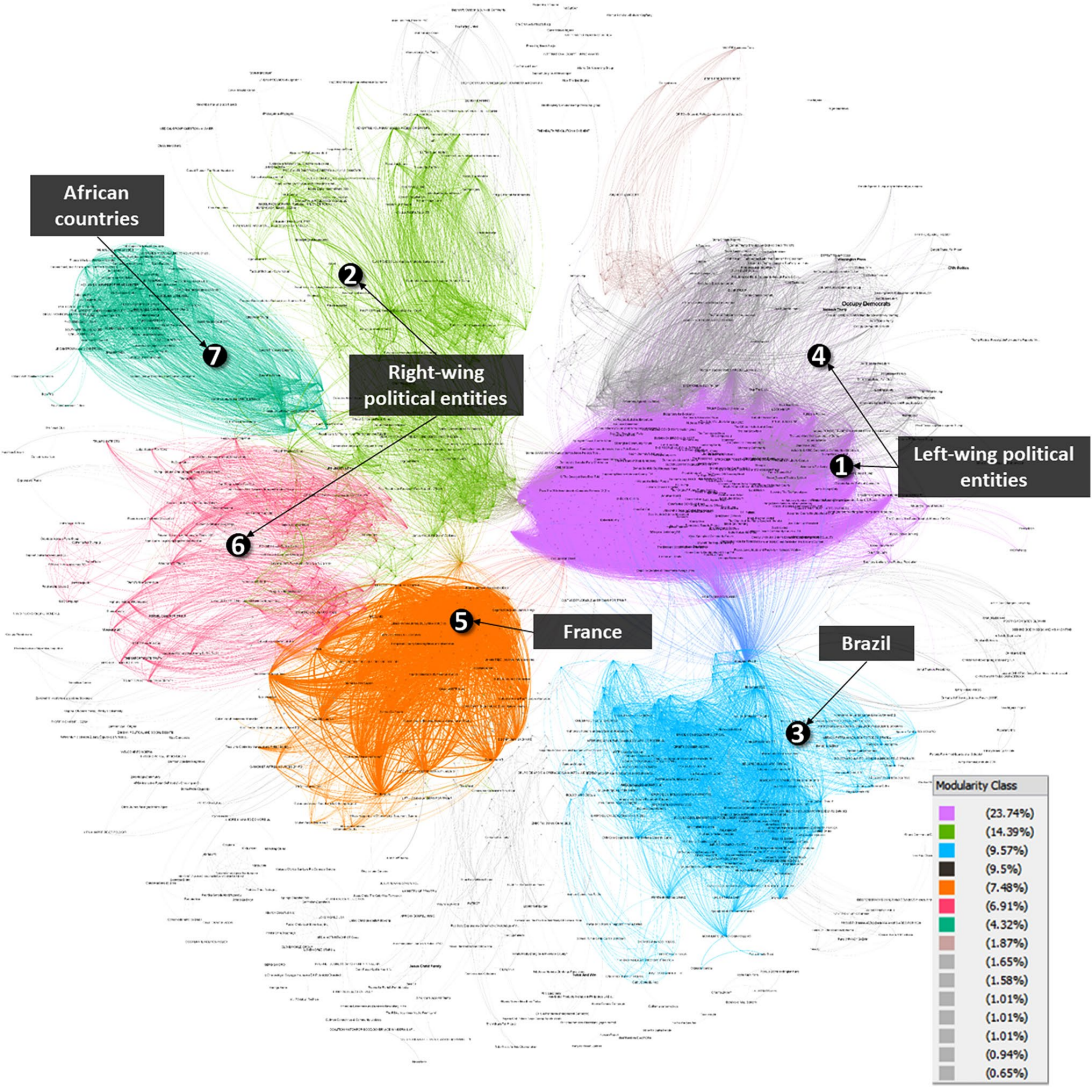
Network of likely coordinated link sharing behavior among 1390 Facebook entities (The percentages in the legend “Modularity Class” represent the percentage of nodes affiliated with each cluster)

Based on the CooRnet analysis, we can positively answer our first research question that, yes, there was clear evidence of CLSB in the collected dataset. Specifically, we identified a network of 1,390 Facebook entities engaged in coordinated link sharing (Fig. 1). Out of the 7,737 public posts shared by these entities as captured in our dataset, 2,484 (36.8%) were shared in a coordinated manner, i.e., they met the 71-s threshold for “unusually” fast sharing, as established by CooRnet.

While connections between Facebook entities in the network are not necessarily a sign of explicit coordination, the discovered linkages reveal clusters of entities that share similar views and thus share and discuss similar links. Furthermore, the fact that these entities share the same links within seconds suggests that these are highly mobilized communities of users who are paying attention to each other and to the news, and whose members are ready and willing to disseminate information or misinformation on a moment’s notice. In the network visualization, these clusters are shown as densely connected groups of nodes and highlighted using different colors. The following Sects. 4.2 and 4.3 will take a closer look at the entities and content shared by Facebook entities found within the seven largest clusters. These seven clusters contained 76% of all nodes in the network.

### What is the role of political polarization and partisan outlets in CLSB? Is CLSB a tactic used by either or both the right- and left-wing political actors on Facebook to spread narrative and counter-narrative in support of their political views?

When we explored the entities that comprise each cluster (as outlined below), the analysis revealed the involvement of groups from both sides of the political divide in the USA. This indicates that CLSB is a tactic used by both right- and left-wing actors.

On the right side of the political spectrum, *Cluster 2* mostly consisted of pro-Trump groups such as “TRUMP 2020 KEEP AMERICA GREAT !”, “Trump's New Generation” and “Donald Trump 2020.” In addition to sharing links to the press conference (live stream or video recording), members of these groups also reposted Fox News coverage of Trump's briefing the day after the “White Coat Summit” press conference, where the President defended the pro-hydroxychloroquine doctor (Re 2020). Another frequently shared source in this cluster was a story about the press conference by the Gateway Pundit (Laila 2020), an extreme right-wing news and opinion website known to share misinformation and conspiracy theories (mediabiasfactcheck.com). *Cluster 6* has formed around groups advocating against mandatory vaccination, QAnon conspiracy theory groups (Wendling 2021), and a fan group of Candace Owens, an influential pro-Trump conservative commentator and political activist. Similar to accounts in Cluster 2, Cluster 6 accounts shared links to the press conference and expressed their support for hydroxychloroquine.

On the left side of the political spectrum, groups in *Cluster 1* and *4* like “Blue Wave 2020,” “EVERYONE HATES TRUMP,” “Joe Biden For President” as well as a number of “Occupy” groups also shared news about the press conference, but mostly citing left-center media outlets like CNN (CNN 2020) and Huffington Post (Robins-Early et al. 2020). These media outlets reported on the event but mostly focused on the danger of promoting health misinformation and President Trump’s reaction to the event. Some articles from more partisan sites like The Daily Beast focused on Dr. Immanuel’s previous “bizarre” claims about other medical topics (Sommer 2020).

To gauge the level of potentially problematic content shared in a coordinated manner within each cluster, we followed the approach used by Yu (2021) and checked how many entities and how many links were no longer available 18 months after the initial data collection. One of the main reasons for accounts and content to disappear is because they shared problematic content. This is largely due to Facebook’s recent efforts to combat pandemic-related misinformation by taking action such as suspending accounts or blocking content in violation of their misinformation policies. In addition, we paid particular attention to whether links that were shared within each of the US-centric clusters led users to partisan media outlets or decentralized platforms, both implicated in helping to spread misinformation and were often used as a method to avoid the platform’s content moderation policies, as noted in the literature review.

Table 1 provides the count and percentage of those Facebook entities that have subsequently become unavailable. The table also recorded whether and how many groups or pages have changed their visibility setting to become private, were renamed, or both. Twenty three percent of right-leaning Facebook entities are no longer available (Clusters 2 and 6), and about the same percentage (20.8%) of Facebook entities on the left side of the political spectrum have also disappeared (Clusters 1 and 4). This is in comparison to 17% of all entities that became unavailable in the whole network. Subject to future research, the similar levels of unavailable entities on both sides of the political spectrum may suggest that Facebook might have been removing US-centric entities engaged in CLSB at about the same rate, irrespective of the content they shared. The percentage of unavailable entities is somewhat smaller in non-US clusters which will be discussed in the next Sect. 4.3.

**Table 1:**
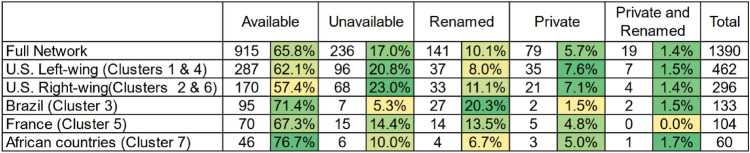
Availability and visibility status of the Facebook entities engaged in CLSB

Colors indicate a higher (green) or lower (yellow) percentage for each category

While the majority of Facebook entities engaged in CLSB on both sides of the political spectrum remained available, we can not say the same about the links they shared. As per the counts presented in Table 2, we identified some key differences in the availability and types of content shared within each of the US-centric clusters. The left-wing entities shared links to both partisan outlets (55.7%) and mainstream media (22%). In contrast, the right-wing entities rarely directed traffic to mainstream media (3.5%), instead most of their posts included links to partisan outlets (34.8%) as well as to social media posts that are no longer available (43.2%). The latter is notable since unavailable content is likely linked to misinformation, as social media platforms worked to remove false and misleading information related to the ‘White Coat Summit’ press conference (Gallagher 2020; Jacqueline 2020). The right-wing entities also linked to the content shared on alternative platforms (6.4%), such as decentralized video sharing sites like BitChute and D.Tube. The strategy to move from mainstream to alternative platforms is often employed by far-right entities that had their content moderated and removed from mainstream platforms (Rauchfleisch and Kaiser 2021).

**Table 2:**
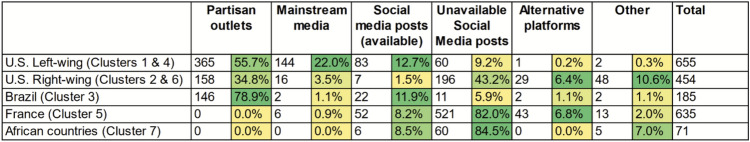
Types and availability of links shared in a coordinated way

The counts in the table refer to the total number of posts that include each type of link, not to the number of unique links in the dataset. Social media posts include posts on Facebook, tweets and videos on YouTube. When we reviewed the links in the dataset, we only identified social media posts that are no longer available. All links to partisan outlets, mainstream media, alternative platforms and other web pages were still active 18 months after the initial data collection

“Other” category includes links that could not be classified in the other categories, such as a link to the ‘White Coat Summit’ website or an academic study related to the use of hydroxychloroquine for COVID-19 treatment. Colors indicate a higher (green) or lower (yellow) percentage for each category

In short, we identified that both right- and left-wing entities in the USA were engaged in CLSB. However, the right-wing entities rarely directed traffic to mainstream media and shared proportionally more links to social media posts that were no longer available (subsequently removed by either the original poster or the platform) than the left-wing entities did.

### Was the CLSB in the studied case constrained to the US-based accounts only? Or, was there a spillover effect, where COVID-19 misinformation originating in the USA was later picked up and shared by Facebook accounts in other countries?

The answer to the final research question is also ‘yes,’ three clusters in the network (Clusters 3, 5, 7) were formed mostly around entities from countries other than the USA. This highlights the fact that we are living in an interconnected world and that social media makes it easier for information and misinformation originating in one country to spread quickly around the world. This is exactly what happened with videos and posts from and about the “White Coat Summit.” Shortly after it was posted online, we see this content was quickly adopted by sympathetic groups in other countries, especially those in Brazil (*Cluster 3*) and France (*Cluster 5*). Some accounts from African countries also engaged in CLSB to spread links about the studied case (*Cluster 7*).

Related to our analysis of Cluster 3, similar to the USA, Brazil had been struggling to reduce the number of new COVID-19 cases when the data was collected in the summer of 2020 (Lovelace Jr 2020). Like in the USA, the country’s leader, President Jair Bolsonaro had been promoting hydroxychloroquine to treat COVID-19, including taking the drug himself (Porterfield 2020). As a result, many pro-Bolsonaro Facebook groups shared the video from the ‘White Coat Summit’ press conference and criticized the left-leaning media for attacking the credibility of the doctors who participated in the press conference. We further explored the links shared by Brazilian entities on Facebook. Since Portuguese is the official language in Brazil, most links shared by entities found in the Brazilian cluster were in Portuguese. This indicates a strategy and a willingness to expend resources to translate and contextualize English content for redistribution in Brazil. Notably, around three-quarters of the posts shared within this cluster linked to stories from Brazilian partisan outlets. In comparison to other clusters in the network, the Brazilian cluster had the fewest unavailable entities (5.3%—Table 1) and links (5.9%—Table 2), 18 months after the initial data collection. Future research is needed to determine factors behind this trend, but one possible explanation is related to the fact that the entities in the Brazilian cluster frequently shared links to external websites (such as partisan outlets), as opposed to other Facebook posts. While Facebook can remove or fact-check native content from the platform, they cannot remove or take down content on external websites.

Based on our analysis of Cluster 5, the press conference has also been shared by many Facebook groups from France. These groups have already been advocating for the use of hydroxychloroquine as a cure for COVID-19 and posting messages in support of French professeur Didier Raoult who was among the first to promote the anti-malaria drug to treat the disease (Sayare 2020). Many of these groups also promoted anti-mask and anti-vaccination policies.

Most of the links shared by entities within the French cluster are no longer available (82%—Table 2). Interestingly, 77.2% of the posts from these entities linked to a single Facebook post containing a video that is no longer available. The French cluster was also the non-US cluster with the most unavailable entities (14.4%—Table 1). Similar to the US right-wing cluster, entities from the French cluster used alternative platforms like D.Tube and BitChute to host videos and avoid the removal of the content (6.8%).

*Cluster 7* identifies similar URL sharing patterns across groups with membership in African countries like Nigeria, South Africa and Cameroon. Posts in this cluster often questioned their government’s response and suggested that their governments are keeping hydroxychloroquine to themselves due to the limited supply to use it as a prophylactic treatment. Similar to the French cluster, most of the posts in this cluster linked to a Facebook post containing an unavailable video (84.5%—Table 2). Additionally, 10% of the entities within this cluster are no longer available on Facebook (Table 1). The available posts in this cluster associate the ‘White Coat Summit’ press conference to local contexts. These included links to a Facebook post from a Nigerian influencer that shared a video of the press conference (8.5%) and to an entertainment website story that highlighted the Cameroonian background of Dr. Stella Immanuel (7%).

## Discussion

Our analysis of rapid link sharing behavior on Facebook reveals a coalition of communities around pro-Trump, QAnon and anti-vaccination groups that are ready to mobilize in unison at a moment's notice. Despite efforts by Facebook and other social media platforms to reactively fact check claims about COVID-19, such groups have been effective at employing a combination of strategies, including CLSB, to propagate conspiracy theories and misinformation in this area. Below is a summary of some of the observed strategies used by these groups which challenge the platforms’ current approaches to managing misinformation around the pandemic (Tangcharoensathien et al. 2020; Zarocostas 2020).

*CLSB was often associated with accounts and content that were later deleted or removed*. Speed is crucial in the process of removing misinformation online. Although a large share of Facebook entities and links are no longer available, entities that engaged in CLSB posted links to problematic content hundreds of times before their removal or deletion. Furthermore, “coordinated” posts received 24.8 shares on average on Facebook (a total of over 61 thousand shares on Facebook). This can create a cascade effect, as posts from coordinated entities are later shared by other Facebook accounts. Therefore, platforms must develop and implement better tools and processes to add friction back into their system to curb abuse associated with rapid, coordinated link sharing to reduce the spread of misinformation online.

*CLSB is a tactic used by both sides of the political spectrum*. While some previous studies found stronger associations between CLSB and right-wing entities (Graham et al. 2020; Yu, 2021), our findings are in line with Nizzoli et al. (2021), who also identified that both right- and left-wing entities engage in coordinated behavior. This indicates that entities from both sides of the political divide use CLSB to push partisan narratives and counter-narratives on social media, which might contribute to increased political polarization. This is particularly problematic in the context of a pandemic when collective and organized action at the societal level is fundamental to reduce the spread of the virus and increase public compliance to protective measures.

*In polarized contexts, misleading claims by country leaders fueled misinformation spread*. Therefore, a challenge faced by social media platforms is when false and misleading claims are propagated by country leaders like President Donald Trump in the USA and President Jair Bolsonaro in Brazil. After all, it is hard to fact check someone who has power to shut down a service. As we saw in our analysis, pro-Trump and pro-Bolsonaro groups were among the strongest contributors in this link sharing network. In particular, the studied case was associated with the discussion about hydroxychloroquine, an unproven drug for COVID-19 that was promoted by both Trump and Bolsonaro (Casarões and Magalhães 2021).

*In addition to political leaders, partisan outlets provided content to mobilize both sides*. On the political right, partisan outlets like Breitbart News reach a receptive audience among political extreme right. On the political left, partisan sites like The Daily Beast shed light on the event while offering narratives to discredit doctors involved in the press conference. Narratives and counter-narratives propagated by partisan sites create an environment where misinformation and distrust strive (Recuero et al. 2020). In such environments, fact checking may not work if it is viewed as a politically motivated tool rather than a health advice (Shin and Thorson 2017).

*In terms of the international reach of misinformation, we identified that what happens in the USA does not stay in the USA.* The strong presence of groups and pages from countries other than the USA shows that many of the groups mentioned above are part of a loosely connected global network of like-minded individuals who are taking a cue from their US counterparts and then using this shared narratives to propel their own agenda in their countries. This means that any action against the spread of COVID-related misinformation on social media has also to be a global response. An example of such a global effort is a series of international meetings and conferences on infodemic hosted by WHO in 2020 and 2021, where practitioners and researchers discussed interdisciplinary approaches to tackling the COVID-19 infodemic around the world (Lancet 2020).

*The Brazilian cluster had the fewest entities and links that were unavailable*. A particular characteristic of this cluster is that most posts are linked to content in Portuguese, particularly stories from Brazilian partisan outlets. The number of unavailable entities from the French and African clusters was also lower compared to the clusters from the USA. As problematic actors are using translation to fuel misinformation spread worldwide (Miguel 2021), platforms ought to find ways to mitigate the influence of problematic content in Portuguese and other non-English languages. Some strategies that we identified include partisan outlets sharing stories about the press conference in Portuguese and videos of Dr. Immanuel’s talk with subtitles in Portuguese and in French. These strategies make the content more accessible to the local population, especially in the case of Brazil with a relatively low English-proficiency in the country (Education First 2021).

*Finally, we identified strategies of decentralization and outsourcing of misinformation*. Despite the efforts by the mainstream social media platforms, many of the videos from the live-streamed press conference remain easily accessible via decentralized platforms like D.tube. This highlights a challenge of combating the spread of COVID-19 misinformation on platforms where there is no single entity that is responsible for curation of such content, and where the platforms often rely on a distribution architecture like blockchain networks designed with replication and anti-deletion policies in mind (Gruzd 2020). Any future action against misinformation must account for these emerging technologies and not just focus on the mainstream social media platforms.

*Study Limitations*. Our study has several limitations that motivate future research and development in this area. First, CrowdTangle, Meta’s platform that we used for data collection, monitors a limited number of entities on Facebook that includes Pages with more than 25 K likes or followers, Groups with 95 K members for non-US-based groups or 2 K members for US-based groups, and all verified profiles (Fraser 2022). Other entities might have been engaged in coordinated behavior. However, we could not identify them due to this data collection limitation.

Second, CrowdTangle does not include the account names of Facebook users that are posted in groups (metadata only includes the name of the group or page). Therefore, we are not able to determine if specific users are posting the same link across different groups (spammers). On top of that, we cannot detect how many CLSB accounts are controlled by multiple or a single entity. Accounts controlled by a single entity (“puppetmaster”) are referred to as “sock puppet” accounts, and are often used to manipulate online discussions (Kumar et al. 2017). To explore this issue, we need stronger collaboration between researchers and platforms, since social media platforms have more data that can be used to detect sockpuppet accounts.

Third, CooRnet, the library we used to detect CLSB, relies on two metadata fields to identify coordination: 1) the link shared and 2) the time difference between posts from different Facebook entities. Although this approach can detect signs of coordination, we cannot necessarily know for sure that all entities identified by CooRnet are coordinating their online action in an explicit manner. This is a limitation just to a certain extent, as partisan actors often follow the same information sources and reshare content as soon as it becomes available. Therefore, they do not need to be in the same room to agree on what and when to share content online. Future research can further explore the notion of coordination in this context.

## Conclusions

Our analysis of posts mentioning Dr. Immanuel revealed that there exists a US-based coalition of Facebook entities consisting of pro-Trump, QAnon and anti-vaccination accounts on Facebook that are acting in concert and engaging in CLSB. These entities frequently shared links to content from conservative news outlets like Fox News and partisan websites like Breitbart News. Much of the content that they link to are to stories that promoted unproven COVID-19 treatments and conspiracies involving COVID-19.

Interestingly, but not surprisingly, our analysis shows CLSB is a tactic also employed by entities on the left side of the political spectrum. Left-wing actors used online discourse around Dr. Immanuel to mobilize their supporters and counter the misinformation. Anti-Trump and pro-Biden pages and groups shared links to news about the danger of promoting health misinformation and President Trump’s reaction to the video involving Dr. Immanuel by left-center media outlets like CNN and Huffington Post. Some also shared articles from more partisan sites like The Daily Beast which focused on Dr. Immanuel’s previous “bizarre” claims about other medical topics (Sommer 2020).

Finally, we discovered a strong presence of groups and pages from Brazil, France and some African countries that also engaged in CLSB in the discourse around Dr. Immanuel. Most of these entities shared links to the video with Dr. Immanuel and expressed pro-hydroxychloroquine sentiments. The presence of non-US entities actively participating in what was essentially a US-centric discussion reinforces the idea that information and misinformation are not bounded by geography. A piece of (mis)information originating in one country can spread around the globe in a matter of minutes, or even seconds. As a result, any action taken to mitigate the spread of COVID-19 related misinformation on social media has to be a global response.

The public has the right to demand a faster response, a better coordination across social media platforms and a speedier fact-checking response, especially when it comes to combating COVID-19 misinformation. The case of the “White Coat Summit” press conference demonstrates that social media platforms are not ready to handle the viral spread of misinformation in highly partisan, internationalized and decentralized information environments. As a result, the “Whac-A-Mole” style approach to combating the spread of misinformation as it propagates across different accounts and platforms is likely to continue, especially in highly polarized countries like the USA and Brazil, countries with a strong presence of influential partisan media and with country leaders whose efforts undermine their health policy advisors and whose actions stand against evidence-based decision making.

## Data Availability

The dataset for this study was collected using CrowdTangle. According to the CrowdTangle data sharing policy, the data is public in nature, but it is only available to organizations and individuals with a CrowdTangle account. Researchers may request access at https://help.crowdtangle.com/en/articles/4302208-crowdtangle-for-academics-and-researchers.
